# Differences in flavonoid pathway metabolites and transcripts affect yellow petal colouration in the aquatic plant *Nelumbo nucifera*

**DOI:** 10.1186/s12870-019-1886-8

**Published:** 2019-06-24

**Authors:** Huan-huan Zhu, Ju-xiang Yang, Chu-han Xiao, Tian-yu Mao, Jie Zhang, Hong-yan Zhang

**Affiliations:** Key Laboratory of Horticultural Plant Biology, Ministry of Education, College of Horticulture and Forestry Sciences, Huazhong Agricultural University, Wuhan, 430070 China

## Abstract

**Background:**

The Asia lotus (*Nelumbo nucifera* Gaertn.) is an ornamental aquatic plant with high economic value. Flower colour is an important ornamental trait, with much of *N. nucifera* breeding focusing on its yellow flowers. To explore the yellow flower colouration mechanism in *N. nucifera*, we analysed its pigment constituents and content, as well as gene expression in the flavonoid pathway, in two *N. nucifera* cultivars.

**Results:**

We performed metabolomic and gene expression analyses in two *N. nucifera* cultivars with yellow and white flowers, Molinqiuse (MLQS) and Yeguangbei (YGB), respectively, at five stages of flower colouration. Based on phenotypic observation and metabolite analyses, the later stages of flower colouration (S3–S5) were determined to be key periods for differences between MLQS and YGB, with dihydroflavonols and flavonols differing significantly between cultivars. Dihydroquercetin, dihydrokaempferol, and isorhamnetin were significantly higher in MLQS than in YGB, whereas kaempferol was significantly higher in YGB. Most of the key homologous structural genes in the flavonoid pathway were significantly more active in MLQS than in YGB at stages S1–S4.

**Conclusion:**

In this study, we performed the first analyses of primary and secondary *N. nucifera* metabolites during flower colouration, and found that isorhamnetin and kaempferol shunting resulted in petal colour differences between MLQS and YGB. Based on our data integration analyses of key enzyme expression in the putative flavonoid pathways of the two *N. nucifera* cultivars, *NnFLS* gene substrate specificity and differential expression of *NnOMT*s may be related to petal colour differences between MLQS and YGB. These results will contribute to determining the mechanism of yellow flower colouration in *N. nucifera*, and will improve yellow petal colour breeding in lotus species.

**Electronic supplementary material:**

The online version of this article (10.1186/s12870-019-1886-8) contains supplementary material, which is available to authorized users.

## Background

The lotus is an economically important aquatic plant that has been widely used for food, medicinal, and ornamental purposes [1–3]. As a basal eudicot plant with numerous monocot characteristics, the lotus also has a special place in evolutionary and taxonomic studies [3, 4]. The lotus also plays a vital role in cultural and religious activities and is extensively distributed throughout Asia and Northern Australia. Two lotus species are recognised based on morphological characteristics: the Asia lotus (*Nelumbo nucifera* Gaertn.) and American lotus (*N. lutea* Pers.) [5–7].

Flower colour is an important trait that determines ornamental quality and landscaping application value. Compared with other ornamental plants, the lotus has not yet been bred to exhibit a wide range of flower colour; Asia lotus cultivars contain only red, pink, and white flowers, whereas the American lotus contains only yellow flowers [8]. Interestingly, there is no reproductive barrier between cultivars of the two lotus species, and more than 800 cultivars with diverse flower colours have been created by natural and artificial hybridisation [9, 10]. Although Asia–American lotus hybrids have produced progeny with yellow flowers, they are very light in colour. Novel yellow cultivars are rare germplasm in lotus breeding, and are precious materials for the study of flavonoid and carotenoid biosynthesis. Thus, a primary goal of lotus breeders is to cultivate new lotus varieties with yellow flowers.

Flower colour is the result of metabolite (pigment) accumulation in the vacuoles of flower epidermal cells [7, 11–13]. Yellow flower colour is thought to be caused by differences in the presence, amount, or type of flavonoid or carotenoid pigments [7]. Yellow flower petals have often been reported to contain carotenoids; yellowish xanthophylls are the main carotenoids in the flower petals of most plants. For example, most carotenoids in the petals of *Sandersonia aurantiaca* are β,β-carotenoids [14]; more than 90% of carotenoids in the petals of marigold (*Tagetes* spp.) [15] and chrysanthemum [16] are lutein and/or lutein derivatives. The accumulation of large amounts of violaxanthin and other carotenoids appears to cause yellow petals in *Brassica napus* [17]. Chalcones and aurone flavonoids are two major target pigments in the flavonoid pathway used to genetically engineer yellow flowers [18]. As intermediates in the biosynthesis of all flavonoids, chalcones have been reported in ornamental species such as carnations (*Dianthus caryophyllus*), cyclamens (*Cyclamen persicum*) and safflower (*Carthamus tinctorius*) as major constituents of yellow flower pigments [19, 20]. Aurone and bright yellow flavonoids have been detected in yellow flowers of dahlia (*Dahlia variabilis*) and snapdragon (*Antirrhinum majus*). Overexpression of the chalcone 4′-O-glucosyltransferase (*4’CGT*) and *AmAS1* genes was associated with accumulation of aureusidin 6-O-glucoside in transgenic *Torenia hybrida* flowers [21]. Flavonols such as kaempferol, quercetin, and isorhamnetin have also been reported to contribute to yellow flower colouration in *Lathyrus chrysanthus* [22], *Camellia chrysantha* [23], *Eustoma grandiflorum* [24, 25] and *Nelumbo nucifera* [7].

To date, few metabolite composition and gene expression analyses of yellow lotus flowers have been performed, with most studies focused on flavonoid pigments. One study compared metabolite content in 108 lotus cultivars with diverse colours, and found that flavones and flavonols were associated with yellow flower colour [7], with isorhamnetin, quercetin, and kaempferol derivatives among the most abundant; in contrast, cultivars with white flowers had higher levels of kaempferol derivatives [7]. These findings were consistent with the lack of anthocyanins detected in lotus cultivars with yellow and white flowers; however, it remains unknown whether yellow lotus flower petals contain carotenoids. Using high-performance liquid chromatography (HPLC), Katori et al. [26] detected lutein and β-carotenoid in lotus cultivars with yellow flowers; however, a study using petroleum ether colour reaction detected no carotenoid pigments in such flowers [27]. Thus, the key metabolites and differential gene expression affecting yellow petal formation in lotus remain unclear. Some studies have examined expression patterns of biosynthetic genes in lotus cultivars with diverse colours. cDNA clones of seven flavonoid biosynthetic genes in four lotus cultivars were isolated, and their expression patterns suggested that in *Nelumbo nucifera* cultivars with different flower colours, flavonoid biosynthesis was differentially regulated by the expression of flavonoid biosynthetic genes, among which *NnCHI*, *NnF3’H*, *NnDFR*, and *NnANS* affected flower colouration [10]. An analysis of the expression patterns of anthocyanin biosynthetic genes and pigments in two lotus cultivars with red and white flowers indicated that *ANS* may be a critical gene conferring anthocyanin accumulation; different methylation intensities on the promoter sequence of the *ANS* gene may result in different flower colouration [12].

Previous studies have mainly focused on improving the methods of extraction and isolation of secondary metabolites in lotus cultivars with red flowers. During flower colouration, the complex network of metabolites and gene expression is dramatically altered [28]. However, little is known about the metabolism and pathway gene expression of representative flower colouration stages, especially for yellow petal colour. Metabolomics is an excellent tool for analysing metabolism during flower colouration, due to its ability to follow metabolic changes dynamically. We therefore selected two lotus cultivars as plant materials: Molinqiuse (MLQS), which has yellow flowers, and Yeguangbei (YGB), which has white flowers. Global metabolomic changes and expression patterns of key flavonoid pathway genes at five determinant flower colouration stages were systematically analysed to elucidate metabolic and transcript differences in the flavonoid pathway. The results of this study will facilitate the determination of the mechanism of yellow flower colour formation in lotus, which is crucial for ornamental lotus breeding.

## Results

### Flower colouration in *N. nucifera* cultivars MLQS and YGB

**Fig. 1:**
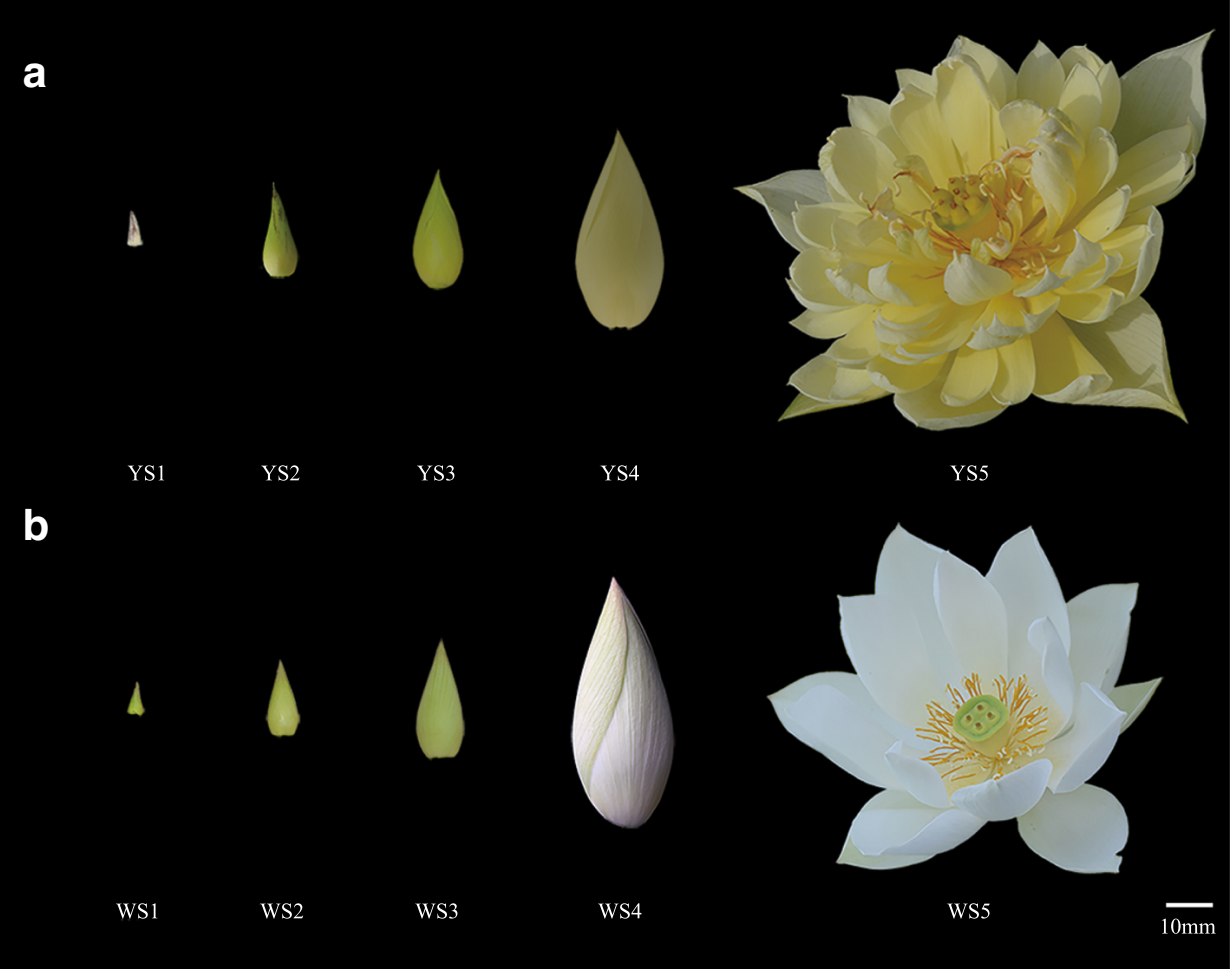
Petal color phenotypes of MLQS and YGB at five representing flower colouration stages. **a** MLQS, defined as Y **b** YGB, defined as W. S1, S2, S3, S4 and S5 represent five different colouration stages

**Fig. 2:**
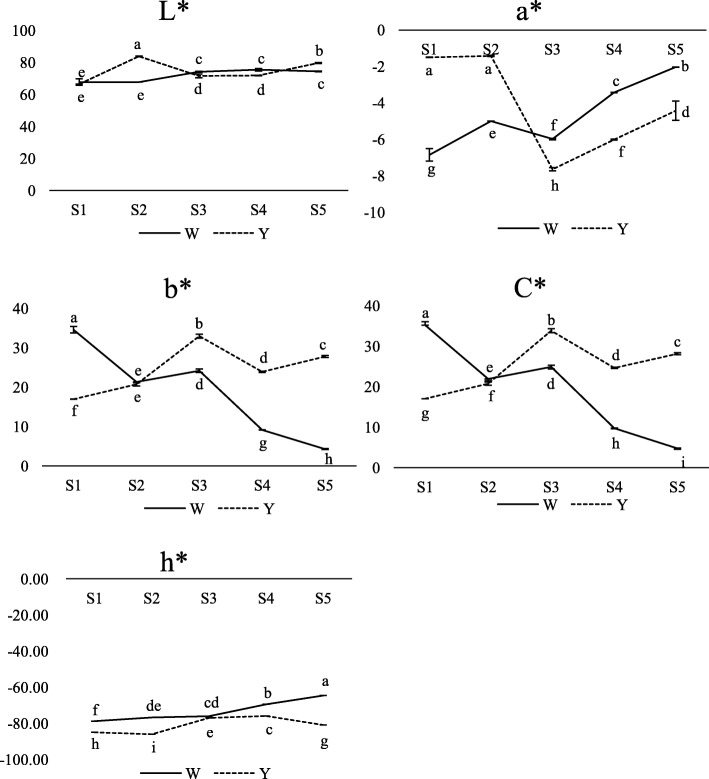
Changes of flower color parameters (L*, a*, b* and C*) of MLQS and YGB at five flower colouration stages. The y-axis scales the mean value of three biological repeats. Lower case letters show significant differences (*P* < 0.05) of the color parameter among stages based on ANOVA and Duncan’s multiple range test

To identify and characterise the developmental stages of lotus flower colouration, we recorded the entire process of flower bud development during 3 consecutive years. We identified five different colouration stages (S1–S5; Fig. 1). In MLQS, yellow colouration deepened throughout S1–S3, reaching a peak in S3, followed by a slight decrease in S4 and an increase in S5. In YGB, yellow colouration peaked in S2, and then gradually faded to white during S3–S5. Changes in flower colour parameters during S1–S5 are shown in Fig. 2, where C* represents chroma, b* represents yellow, and positive values indicate trends consistent with phenotypic observations. C* and b* generally showed a decreasing trend in YGB, whereas in MLQS, they showed an increasing trend, reaching a first peak at S3 and a second peak at S5. L* remained constant after a slight increase during S2–S3 in YGB; in MLQS, it increased rapidly during S1–S2, then decreased, and increased slightly at S5. a* showed opposite trends in MLQS and YGB at the early and late stages of flower colouration; a* was larger in MLQS than in YGB in the early stages, and smaller in the later stages. However, a* generally increased in both MLQS and YGB during S3–S5. h* values were closer to 90u (yellow) in MLQS than in YGB (Fig. 2).

### Primary metabolic profiling of MLQS and YGB during flower colouration

Representative petals of the two selected lotus varieties were collected at S1–S5 and used for metabolite extraction followed by GC–MS analysis [29]. A total of 46 metabolites were identified using an available chromatogram library (Additional file 2: Table S2). The content of each metabolite detected at each flower colouration stage is provided in Additional file 3: Table S3.

**Fig. 3:**
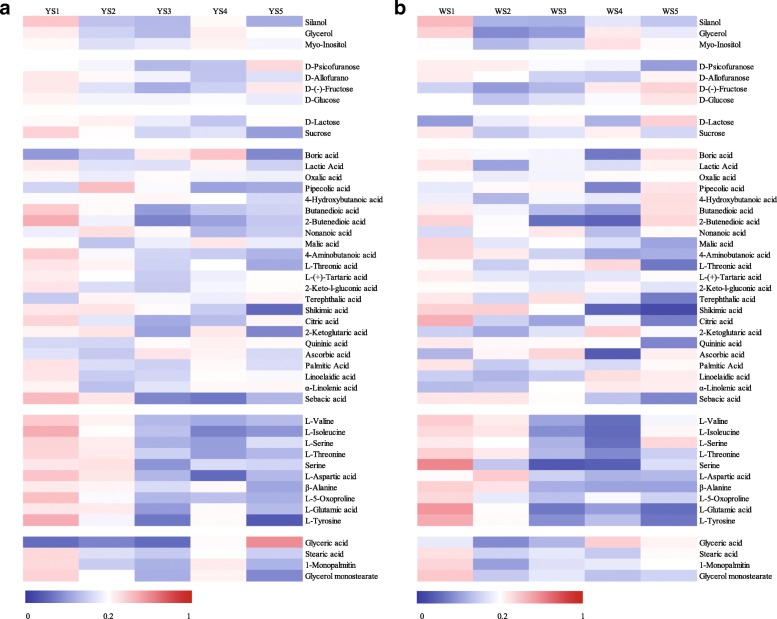
Heat map showing dynamic changes of primary metabolites during flower colouration of **a** MLQS and **b** YGB. The proportion of each metabolites in all periods from minimal to maximum are colored from blue to red

The dynamics of sugar and organic acid metabolism differed between MLQS and YGB (Additional file 3: Table S3). Nearly all sugars were significantly reduced at S1–S2 in MLQS and YGB flowers (*P* < 0.05). In the later stages, especially S5, D-allofuranose, D-glucose, and D-lactose content increased significantly in YGB, whereas D-allofuranose and D-glucose content decreased in MLQS. D-psicofuranose content increased significantly in S5 in MLQS, but decreased significantly in YGB (Additional file 3: Table S3; Fig. 3). Patterns in organic acid content also differed between MLQS and YGB, especially at the later stages of flower colouration (Fig. 3). In MLQS, around half of the organic acids showed decreasing trends. Our analysis of ANOVA and Duncan’s multiple range test results revealed that terephthalic acid, citric acid, and quininic acid decreased significantly in YGB at S5, whereas citric acid content increased significantly in MLQS at S5 (Additional file 3: Table S3). Boric acid, lactic acid, and palmitic acid content showed opposite patterns between MLQS and YGB (Fig. 3). The content of these organic acids was significantly higher in S4 than in S3, and significantly lower in S5 than in S4 in MLQS, whereas the opposite pattern was observed in YGB (Additional file 3: Table S3). Similar patterns in amino acid, glyceric acid, and glycol content were observed between MLQS and YGB during flower colouration, with some metabolites differing significantly at the later stages (Fig. 3; Additional file 3: Table S3). Amino acid content showed a similar decreasing trend in MLQS and YGB, with the highest content detected at S1 or S2 (Fig. 3). In contrast, L-valine, L-serine, and L-isoleucine contents were significantly higher in S5 than in S4 in YGB, whereas in MLQS, only L-serine and L-aspartic acid contents were significantly higher in S5 (Additional file 3: Table S3).

Pearson correlation coefficients were calculated for comparisons of b* values between MLQS (Y) and YGB (W) to evaluate the primary metabolites of Y/W during the five flower colouration stages (Table 1). Based on these correlation analyses, most primary metabolites were negatively correlated with Y/W b* values, except for some organic acids and amino acids. Among these, D-psicofuranose, terephthalic acid, citric acid, quininic acid, and glyceric acid showed significant positive correlation with Y/W b* values (*P* < 0.05), whereas D-allofuranose and D-glucose were significantly negatively correlated with Y/W b* values (Table 1). Interestingly, primary metabolites significantly correlated with Y/W b* values also exhibited different dynamics between MLQS and YGB, as described above (Fig. 3; Additional file 3: Table S3).

**Table 1:** Pearson correlation analyses between the metabolites and the b* value of Y/W

Metabolites	R	R^2^	p
Primary metabolites			
Silanol	−0.440	0.194	0.459
Glycerol	−0.108	0.012	0.863
Myo-Inositol	−0.656	0.430	0.229
D-Psicofuranose	0.940*	0.884	0.018
D-Allofuranose	−0.923*	0.852	0.026
D-(−)-Fructose	−0.621	0.386	0.263
D-Glucose	−0.964**	0.929	0.008
D-Lactose	−0.712	0.507	0.178
Sucrose	−0.749	0.561	0.145
Boric acid	−0.038	0.001	0.952
Lactic acid	−0.534	0.285	0.354
Oxalic acid	−0.587	0.345	0.298
Pipecolic acid	−0.581	0.338	0.305
4-Hydroxybutanoic acid	−0.808	0.653	0.098
Butanedioic acid	−0.708	0.501	0.181
2-Butenedioic acid	−0.495	0.245	0.397
Nonanoic acid	−0.668	0.446	0.218
Malic acid	0.810	0.656	0.097
4-Aminobutanoic acid	0.221	0.049	0.721
L-Threonic acid	0.277	0.077	0.652
L-(+)-Tartaric acid	−0.242	0.059	0.695
2-Keto-l-gluconic acid	0.262	0.069	0.671
Terephthalic acid	0.916*	0.839	0.029
Shikimic acid	0.687	0.472	0.200
Citric acid	0.906*	0.821	0.034
2-Ketoglutaric acid	−0.627	0.393	0.258
Quininic acid	0.997**	0.994	0.000
Ascorbic acid	−0.018	0.000	0.977
Palmitic acid	−0.436	0.190	0.463
Linoelaidic acid	−0.666	0.444	0.219
α-Linolenic acid	−0.461	0.213	0.435
Sebacic acid	0.314	0.099	0.607
L-Valine	−0.255	0.065	0.679
L-Isoleucine	−0.675	0.456	0.211
L-Serine	−0.648	0.420	0.237
L-Threonine	−0.396	0.157	0.510
Serine	−0.136	0.018	0.828
L-Aspartic acid	−0.238	0.057	0.700
β-Alanine	0.160	0.026	0.798
L-5-Oxoproline	−0.688	0.473	0.199
L-Glutamic acid	0.783	0.613	0.117
L-Tyrosine	−0.273	0.075	0.657
Glyceric acid	0.925*	0.856	0.024
Stearic acid	−0.510	0.260	0.380
1-Monopalmitin	−0.430	0.185	0.470
Glycerol monostearate	−0.355	0.126	0.557
Secondary metabolite			
Phenylalanine	0.657	0.432	0.228
Coumaric acid	−0.587	0.345	0.298
Dihydrokaempferol(−D)	−0.560	0.314	0.327
Dihydromyricetin	0.956*	0.914	0.011
Dihydroquercetin	0.468	0.219	0.427
Kaempferol(−D)	−0.517	0.267	0.373
Myricetin(−D)	0.815	0.664	0.092
Quercetin(−D)	0.931*	0.867	0.022
Catechin(−D)	0.874	0.764	0.053
Isorhamnetin(−D)	0.972**	0.945	0.006

Note: * indicates significant correlation at *P* < 0.05 level, with ** indicate highly significant correlation at *P* < 0.01 level

### Secondary metabolic profiling of MLQS and YGB during flower colouration

**Fig. 4:**
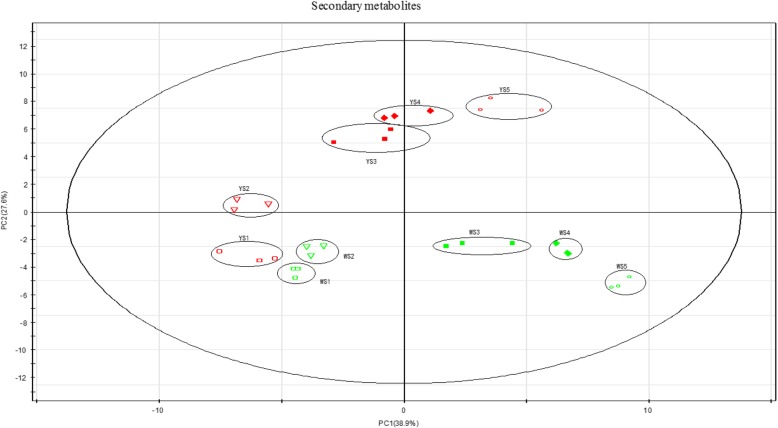
Principal component analysis of LC-MS metabolite profiling data

We explored our LC–MS results using principal component analysis (PCA) to detect differences in major secondary metabolites between the two lotus cultivars. Two PCs were calculated by cross validation; 38.9% of the variation was explained by the first component and 27.6% by the second component (Fig. 4). A clear secondary metabolic shift was observed in both MLQS and YGB from the earlier to the later flower colouration stages, when the yellow petal colour of MLQS gradually deepened. The earlier flower colouration stages of both lotus cultivars were grouped together, but separate from the later stages (Fig. 4). These PCA analysis results were consistent with the phenotypic characteristics and b* values of the two lotus cultivars (Figs. 1 and 2). A total of 78 metabolites were detected, including phenylalanine, coumaric acid, dihydroflavonols, flavonols, and their derivatives (Additional file 4: Table S4).

**Fig. 5:**
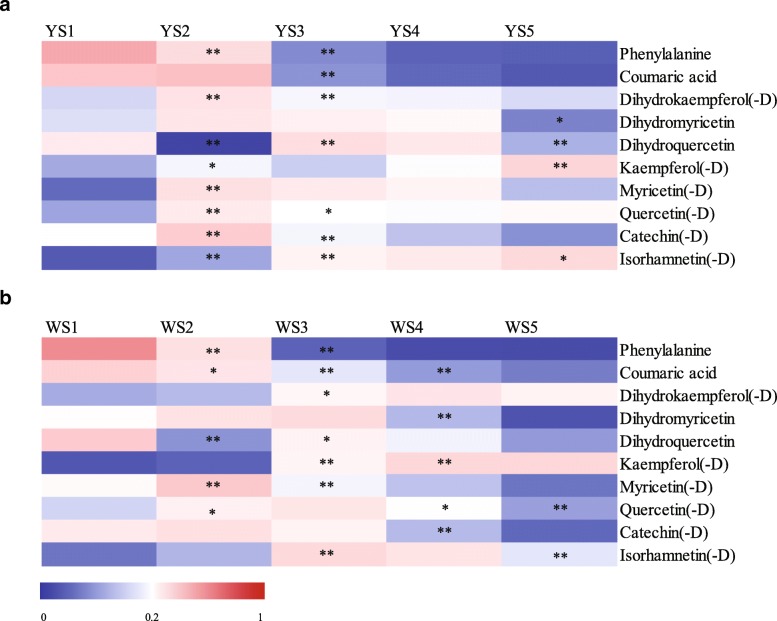
Heatmap showing dynamic changes of the 10 secondary metabolites and their derivatives in **a** MLQS and **b** YGB during flower colouration. The proportion of each secondary metabolites and their derivatives in all periods from minimal to maximum are colored from blue to red. * indicate significant differences (*P* < 0.05) with ** indicate highly significant differences (*P* < 0.01) between MLQS and YGB

Both lotus cultivars generally displayed similar accumulation patterns among the 10 secondary metabolites and their derivatives during the flower colouration process. However, significant differences in flavonol content were detected from S1 to S5 (Fig. 5). Variations in phenylalanine, coumaric acid, dihydromyricetin, myricetin(−D) (the total content of metabolite and its derivatives), catechin(−D), and dihydroquercetin content were similar from S1 to S5, with more consistent variation in phenylalanine and coumaric acid content detected in both MLQS and YGB, decreasing continuously from S1 to S5 (Fig. 5). Dihydromyricetin, myricetin(−D), and catechin(−D) content was first accumulated, and then consumed (Fig. 5). Dihydroquercetin content fluctuated with flower colouration, decreasing in both MLQS and YGB at the later stages and decreasing significantly during S4–S5 in YGB. The variation trend of dihydrokaempferol(−D) content differed between cultivars, increasing significantly in MLQS at the earlier stages, decreasing significantly at S2–S3, then decreasing slightly thereafter. In YGB, dihydrokaempferol content increased at S1–S4, and then decreased slightly at S4–S5.

Except for similar myricetin(−D) content, flavonols (isorhamnetin, quercetin, and kaempferol) and their derivatives differed distinctly in content between MLQS and YGB. Isorhamnetin(−D) was significantly accumulated in a nearly continuous manner in the yellow cultivar MLQS (Fig. 5). However, in YGB, they increased only during S1–S3, and decreased significantly during S4–S5 (Fig. 5). Quercetin(−D) content increased significantly in both MLQS and YGB at the earlier stages of flower colouration, and significantly decreased from S3 to S5 in YGB; in MLQS it decreased significantly only during S2–S3. Kaempferol(−D) content remained constant in YGB, but increased significantly in a fluctuating manner from S2–S4 in MLQS.

Variation trends in the content of the 78 secondary metabolites were similar to those of the 10 secondary metabolites and their derivatives (Fig. 5). Flavonol content varied quite differently between MLQS and YGB (Additional file 5: Figure S1; Additional file 6: Table S5). Concentrations of isorhamnetin and its derivatives increased significantly throughout the five stages in MLQS, whereas they decreased significantly during the later flower colouration stages in YGB (Additional file 6: Table S5). Quercetin and its derivatives showed similar content variation trends to quercetin(−D), with more quercetins decreasing significantly in content at the later stages in YGB (Additional file 6: Table S5). Some kaempferol derivatives showed different variation trends from kaempferol(−D). For example, kaempferol derivatives 4, 7, 8, 11, and 17 showed downward trends in YGB during the later flower colouration stages that were similar to those of kaempferol(−D) in MLQS (Additional file 6: Table S5).

Pearson correlation coefficients were used to assess the relationship between flower colour and major secondary metabolites (Table 1). Most metabolites were positively correlated with Y/W b* values, except coumaric acid, dihydrokaempferol(−D), and kaempferol(−D). Among these, dihydromyricetin and quercetin(−D) were significantly positively correlated with Y/W b* (*P* < 0.05), and isorhamnetin(−D) was highly significantly correlated (*P* < 0.01).

### Significantly differential metabolites analyses between MLQS and YGB

**Fig. 6:**
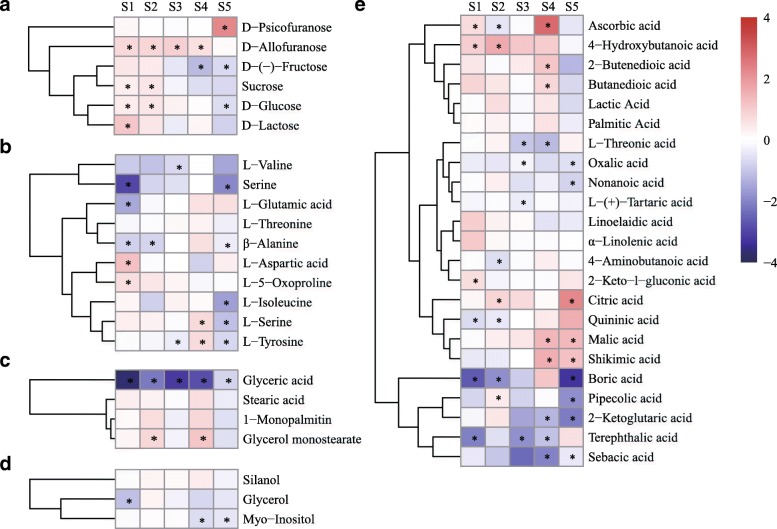
Difference between MLQS and YGB on primary metabolites during flower colouration. Heatmaps represent log_2_FC(Y/W) of the **a** sugars, **b** amino acids, **c** fatty acids, **d** alcohols, and **e** organic acids. Red and blue indicate comparing with YGB, MLQS increased or decreased. * indicate significant differences (P < 0.05) between MLQS and YGB

There were 17, 13, 8, 16, and 19 significantly different primary metabolites between MLQS and YGB at each of the five flower colouration stages, respectively (Fig. 6). More primary metabolites had significantly higher content in MLQS than in YGB during S1, S2, and S4, and more primary metabolites had significantly lower content in MLQS than in YGB during S3 and S5 (*P* < 0.05). Differences in sugar, organic acid, amino acid, and fatty acid content between MLQS and YGB were diverse among the flower colouration stages (Fig. 6), but metabolite content was generally significantly lower in MLQS than in YGB.

Specifically, the content of most sugars was significantly higher in MLQS than in YGB during S1 and S2 (*P* < 0.05). However, the opposite trend was observed in S4 and S5, with lower content of most sugars in MLQS, except for D-psicofuranose (Fig. 6a). Five amino acids were significantly different between MLQS and YGB at S1 and S5, and amino acid content differed slightly between cultivars at the other three stages (Fig. 6b). Content of organic acids including boric acid, 2-ketoglutaric acid, terephthalic acid, and sebacic acid, was significantly lower in MLQS than in YGB during most of the flower colouration stages. Conversely, malic acid, shikimic acid, and citric acid content were significantly higher in MLQS than in YGB at the later stages (Fig. 6e). Among the four fatty acids detected, glyceric acid content was significantly lower in MLQS than in YGB from S1 to S5 (Fig. 6c), whereas glycol content was similar between cultivars (Fig. 6d).

**Fig. 7:**
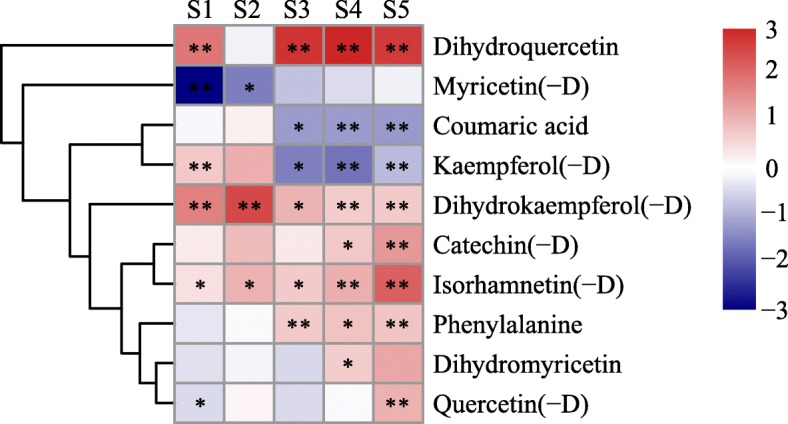
Difference between MLQS and YGB on the 10 secondary metabolites and their derivatives during flower colouration. Heatmaps represent log_2_FC(Y/W) of the sum of secondary metabolites and derivatives. Red and blue indicate comparing with YGB, MLQS increased or decreased. * indicates significant differences (P < 0.05) between MLQS and YGB, and ** indicates highly significant differences (*P* < 0.01)

Among the 10 secondary metabolites and their derivatives, most were significantly higher in MLQS than in YGB regardless of flower colouration stage (*P* < 0.05; Fig. 7). More secondary metabolites were present in higher concentrations in MLQS as flower colouration progressed. At S4 and S5, the contents of most secondary metabolites (e.g., dihydroquercetin, dihydrokaemferol, isorhamnetin, and their derivatives) were significantly higher in MLQS than in YGB. At the later stages of flower colouration, the largest difference in secondary metabolite content between cultivars was observed for dihydroquercetin, followed by isorhamnetin(−D). In contrast, concentrations of kaempferol and its derivatives were significantly lower in MLQS at the later stages, which are the key stages of colour differentiation between MLQS and YGB (Fig. 7). Concentrations of phenylalanine, which is an upstream metabolite of the flavonoid pathway, were significantly higher in MLQS than in YGB at the later stages, and those of coumaric acid were significantly lower. Quercetin(−D) content was significantly higher in MLQS only at S5. These results are consistent with the finding that secondary metabolite content was significantly correlated with b*.

A detailed comparison of the secondary metabolites and their derivatives is provided in Additional file 7: Table S6. The entire flavonoid metabolic pathway was more active in MLQS than in YGB, especially at the later stages (S3–S5). Metabolites with significantly higher content in MLQS were very abundant during these stages. There were 43, 24, 34, 42, and 55 significantly different metabolites between MLQS and YGB in S1, S2, S3, S4, and S5, respectively (*P* < 0.05), of which 21, 21, 16, 25, and 45 were significantly higher in MLQS. These results were consistent with our analysis of 10 secondary metabolites and their derivatives (Fig. 7).

To further confirm whether total flavonoid production or the proportion of each substance is important for yellow flower colouration in lotus, we performed Pearson correlation analyses of Y/W secondary metabolite content and Y/W b* values. Isorhamnetin(−D) proportions were highly significantly correlated with b* (*P* = 0.014; R = 0.948); however, total Y/W flavonoid content and b* were not significantly correlated (*P* = 0.445; R = 0451). Thus, the proportion of isorhamnetin(−D) appears to be more important for yellow flower colour than for white flower colour.

### MLQS and YGB flavonoid pathway gene expression profiling

**Fig. 8:**
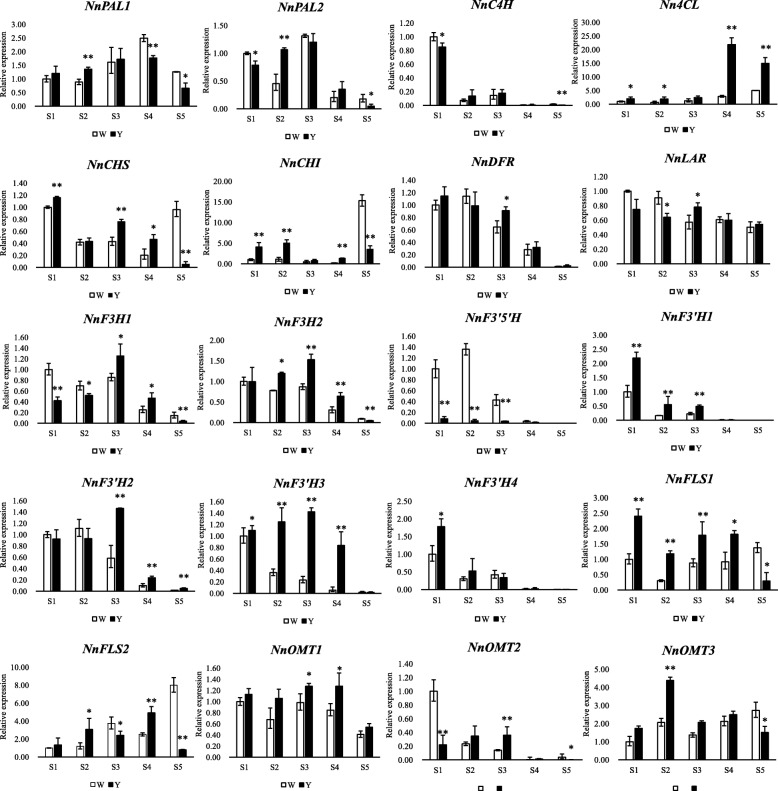
qRT-PCR results of flavonoid pathway structural genes in MLQS and YGB during flower colouration. The y-axis scales the mean value of three biological repeats. * indicates significant differences (*P* < 0.05) between MLQS and YGB, and ** indicates highly significant differences (*P* < 0.01)

To determine whether flavonoid compound expression levels were correlated with mRNA abundance, we performed qRT-PCR analysis. The genes encoding key flavonoid pathway enzymes were analysed at five stages of flower colouration (Fig. 8).

The expression of *NnPAL*, *Nn4CL*, *NnF3H*, *NnF3’H2*, *NnF3’H3*, *NnFLS2*, *NnOMT1*, and *NnOMT2* in MLQS began to increase from the first stage, peaked in S3 or S4, and then decreased in S5 (Fig. 8). This result is consistent with higher secondary metabolite accumulation in MLQS (Additional file 5: Figure S1). However, the expression of *NnC4H*, *NnDFR*, *NnF3’5’H*, *NnF3’H1*, and *NnF3’H4* decreased from S1, reaching a minimum in S5. In YGB, the expression of *NnC4H*, *NnLAR*, and *NnOMT2* also decreased from S1, reaching a minimum in S5. *Nn4CL* expression increased throughout the flower colouration process.

The qRT-PCR results showed that most structural genes in the flavonoid pathway of MLQS were more active than those of YGB at S1–S4, whereas the opposite trend was observed at S5. There were 7, 10, 11, 10, and 2 genes with significantly higher expression levels in MLQS than in YGB during the five stages, respectively (*P* < 0.05). The expression levels of *Nn4CL* and *NnF3’H* were significantly higher in MLQS than in YGB nearly throughout S1–S5, with 14.8-fold and 6.1-fold higher *NnF3’H3* expression at S4 and S3, respectively. *Nn4CL* expression was 7.7-fold higher in MLQS than in YGB at S4. *NnCHS*, *NnCHI*, *NnFLS*, *NnOMT1*, and *NnOMT3* expression was also significantly higher in MLQS than in YGB at S1–S4. Among these genes, *NnFLS1* expression was 3.9-fold higher in MLQS than in YGB at S2, and *NnOMT2* expression was 3.2-fold higher at S4; however, their expression levels were significantly higher in YGB than in MLQS during S5 (Fig. 8).

### Correlation of metabolites and biosynthesis gene expression in MLQS and YGB

We next performed correlation analysis of Y/W primary and secondary metabolites during flower colour formation to evaluate metabolite network behaviour in greater detail. Of the 460 pairs of metabolites analysed, 48 resulted in significant correlations (*P* < 0.05). Many sugars and organic acids were significantly correlated with secondary metabolites, whereas only three amino acids (L-isoleucine, L-5-oxoproline, and L-glutamic acid) and one fatty acid (glyceric acid) were significantly correlated with secondary metabolites (Additional file 8: Table S7). Isorhamnetin(−D), myricetin(−D), and quercetin(−D) had the largest numbers of significantly correlated primary–secondary metabolite pairs. Isorhamnetin(−D) and quercetin(−D) were highly significantly correlated with sugars such as D-allofuranose (R = − 0.902; R = − 0.897) and D-psicofuranose (R = 0.960; R = 0.954), and isorhamnetin(−D) was significantly negatively correlated with D-glucose (R = − 0.882). Three organic acids (terephthalic acid, citric acid, and quininic acid) and one fatty acid (glyceric acid) were significantly positively correlated with isorhamnetin(−D) and quercetin(−D), whereas L-isoleucine was significantly negatively correlated with quercetin(−D) (R = − 0.894). Kaempferol(−D) was significantly correlated with D-(−)-fructose, sucrose, nonanoic acid, and 2-ketoglutaric acid (Additional file 8: Table S7).

**Fig. 9:**
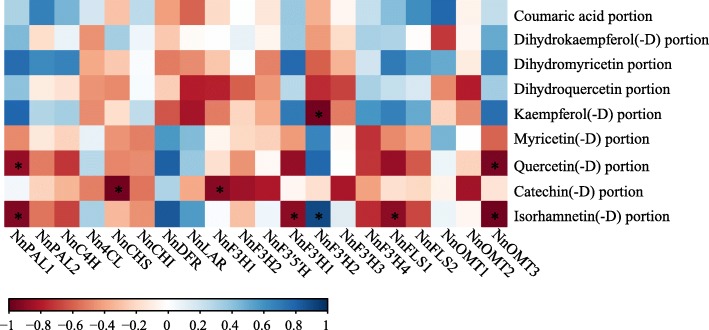
Pearson correlation analysis of the key biosynthetic genes and flavonoids. Red to blue shades represent the degree of positive and negative correlations, respectively. * indicates significant correlation at *P* < 0.05 level

As mentioned above, Y/W flavonoid proportion was significantly correlated with b*. Pearson correlation analysis was conducted to determine the association between key biosynthetic genes and flavonoids (Fig. 9), and showed that correlated genes were nearly identical among isorhamnetin(−D), quercetin(−D), and kaempferol(−D). We detected significant correlations between gene expression and flavonoid proportion in 10 gene pairs (*P* < 0.05). Isorhamnetin(−D) was significantly correlated with *NnPAL1*, *NnF3’H1*, *NnF3’H2*, *NnFLS1*, and *NnOMT3* expression, whereas quercetin(−D) was significantly correlated with *NnPAL1* and *NnOMT3* expression. Kaempferol(−D) was significantly correlated with *NnF3’H2* expression.

## Discussion

### Primary metabolic dynamics during YGB and MLQS flower colouration

Flower colour is an important characteristic for ornamental plants. Although metabolite diversity is a key component of flower colouration, few studies have been conducted on metabolite dynamics during flower colouration in lotus. Compared with other ornamental plants, lotus flowers do not have a wide range of flower colour [8]. In the current study, we used a GC–MS platform to quantify 46 primary metabolites including sugars, amino acids, organic acids, glyceric acids, and alcohols (Additional file 2: Table S2), which are reported to act as chemical precursors and energy for floral secondary metabolism [30].

The results of our content dynamics and differential metabolite content analyses suggested that most sugars were present in higher quantities in MLQS than in YGB during the earlier flowering colouration stages, and in lower quantities at the later stages (Fig. 6). Levels of D-allofuranose, D-glucose, and D-lactose decreased in MLQS and increased significantly in YGB (Fig. 3; Additional file 3: Table S3). D-glucose content was significantly lower in MLQS than in YGB at S5. D-allofuranose and D-glucose content were significantly negatively correlated with Y/W b* (Table 1). Sugars are important in the primary metabolome, as they provide energy resources and carbon skeletons for subsequent metabolic activities, and also work as signalling molecules regulating pigment-related genes at the transcriptional and post-translational levels [31–36]. Glucose has been reported to induce *PsCHS1* and *PsCHI1* expression through glucose signalling in *Paeonia suffruticosa* [37]. Most sugars were significantly higher in content in MLQS at the earlier stages of flower colouration, which may be related to abundant secondary metabolites in MLQS at the later stages. Glycosylation modification is the most common modification of natural flavonoids [38], and glucose, galactose, rhamnose, and arabinofuranose are the most common sugars [38–41]. Interestingly, lower sugar content was detected in MLQS at the later stages.

Different content dynamics (Fig. 3) and metabolite patterns (Fig. 6) were observed among the 23 organic acids detected in MLQS and YGB. Boric acid, 2-ketoglutaric acid, terephthalic acid, and sebacic acid were significantly lower in MLQS than in YGB during most of the flower colouration stages (*P* < 0.05), and malic acid, shikimic acid, and citric acid were significantly more abundant in MLQS at the later stages. Terephthalic acid, citric acid, and quininic acid abundance was positively correlated with Y/W b* (Table 1). To date, few studies have examined the effect of organic acids on flower colour formation. Organic acids including citrus and malic acids have been reported to stabilise flavonoids through acylation [12, 42–45]; however, these acids are intermediates of the tricarboxylic acid (TCA) cycle, which provides energy for metabolism [46]. The significantly higher content of malic acid, shikimic acid, and citric acid observed in MLQS at the later stages may indicate a more active secondary metabolism at later flower colouration stages, which would be consistent with our Pearson’s correlation analysis results.

### Differences in secondary metabolism between cultivars during flower colouration

Previous studies have found that flavonoids are the main pigments in yellow lotus petals. Xu et al. [27] reported that yellow lotus varieties contained no carotenoids; however, Katori et al. [26] detected lutein and β-carotenoid in lotus by applying HPLC, and Deng et al. [7] detected flavonoid pigments in lotus cultivars with yellow flowers. In this study, flavonoid and carotenoid contents in MLQS and YGB at S5 were determined in a preliminary experiment; in MLQS, total carotenoid content was 68.25 μg g^− 1^ dry weight (DW) and total flavonoid content was 401.53 μg g^− 1^ DW, nearly 6-fold higher.

We then focused on the detection of metabolites in the flavonoid pathway. A total of 78 secondary metabolites were detected in LC–MS (Additional file 4: Table S4). PCA analysis indicated a clear secondary metabolic shift from the earlier flower colouration stages to the later stages, with the earlier stages of both cultivars grouped together and separate from the later flower colouration stages (Fig. 4). This result suggests that S3–S5 are the key stages for flower colour differentiation between these lotus cultivars (Fig. 1).

As shown in Additional file 6: Table S5), secondary metabolites in the lotus cultivars comprised phenylalanine, coumaric acid, dihydroflavonols, flavonols, and their derivatives. No anthocyanin, aurone, or chalcones were detected in our analyses, which is consistent with previous studies that found almost no anthocyanins, aurone, or chalcones in yellow and white lotus varieties [10]. In this study, quercetin, isorhamnetin, kaempferol, and their derivatives were the most abundant secondary metabolites in MLQS, whereas kaempferol, quercetin, catechin, and their derivatives were most abundant in YGB (Additional file 6: Table S5). A comparison of secondary metabolites between the two cultivars clearly showed that the entire flavonoid metabolic pathway was more active in MLQS, especially at the later developmental stages (Additional file 7: Table S6; Fig. 7). Dihydroquercetin, dihydrokaempferol(−D), and isorhamnetin (−D) were significantly higher in MLQS than in YGB at almost all flower colouration stages, whereas kaempferol(−D) content was significantly lower in MLQS at the later flower colouration stages (Fig. 7). However, our content dynamics analysis results suggest that dihydroquercetin(−D) and dihydrokaempferol(−D) content decreased significantly at later stages in MLQS (Additional file 6: Table S5). Unsurprisingly, isorhamnetin(−D) content significantly increased at later flower colouration stages and was highly correlated with b* (*P* < 0.05, R = 0.972). The significant correlation of metabolite content with b* was consistent with the results of our metabolite analyses; kaempferol derivatives were most abundant in YGB, consistent with the results of Deng et al. [12], and significantly less abundant than in MLQS at the later stages. We therefore speculate that the process of flavonol synthesis has differentiated in the yellow cultivar MLQS and white cultivar YGB. The Y/W proportion of isorhamnetin(−D) was highly significantly correlated with Y/W b*; however, total flavonoid content and Y/W b* were not significantly correlated, suggesting that the proportion of isorhamnetin(−D) is more important for yellow flower development. Indeed, isorhamnetin(−D) has been reported to be a yellow pigment [47, 48].

Correlation analyses between primary and secondary metabolites showed that 48 of the 460 total metabolite pairs were significantly correlated (*P* < 0.05). Many sugars and organic acids were significantly correlated with secondary metabolites, whereas only three amino acids (L-isoleucine, L-5-oxoproline, and L-glutamic acid) and one fatty acid (glyceric acid) were significantly correlated with secondary metabolites (Additional file 8: Table S7). Due to the lack of a sufficient metabolite standard, the results of our analysis of metabolite derivatives are unclear. Subsequent studies on the derivatives of key metabolites will better reveal the relationship between primary and secondary metabolites in relation to flower colour.

### Differential flavonoid pathway metabolism gene expression analysis

**Fig. 10:**
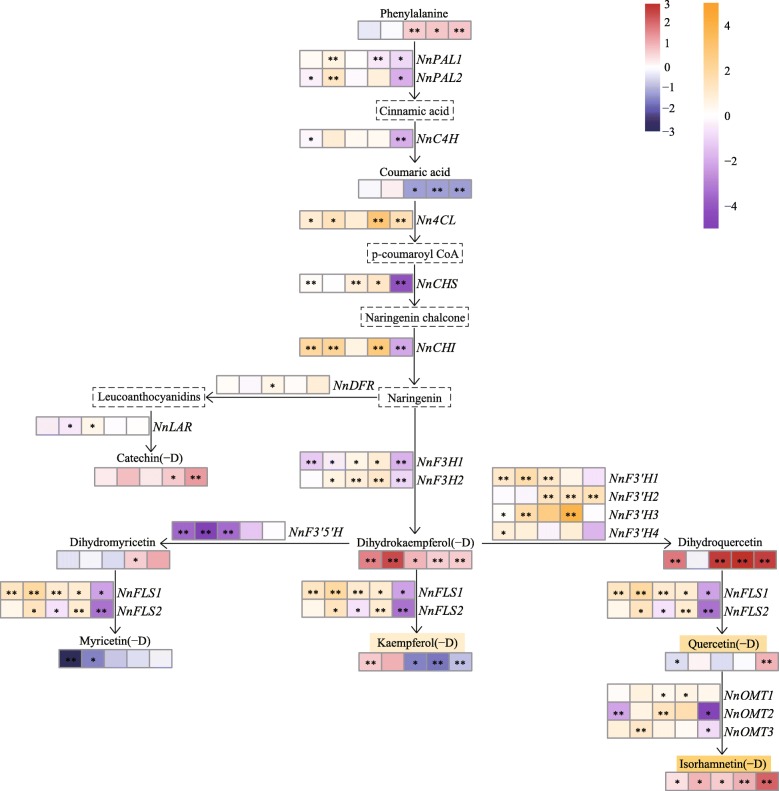
Schematic flow chat of the flavonoid pathway of lotus. Red and blue shades indicate up and down regulated metabolites, orange and purple indicate up-regulated and down- regulated genes, in MLQS compared to YGB at five stages (S1: left1 cell, S2: left2 cell, S3: central cell, S4: right2 cell, S1: right1cell). Box with a dotted line represents the undetected metabolite

Based on the flavonoids detected in MLQS and YGB, we produced a schematic flowchart of the flavonoid pathway relevant to lotus flower colour (Fig. 10). In total, 20 key homologous genes in the flavonoid pathway were detected under strict selection. The overall expression levels of early biosynthetic genes (EBGs) in the flavonoid metabolic pathway (*NnCHS*, *NnCHI*, and *NnF3H*) [49, 50] were higher in MLQS than in YGB at S1–S4, resulting in significant differences in dihydroflavonol between MLQS and YGB. In particular, dihydroquercetin content in MLQS reached 6–8 times that in YGB (Fig. 10). It has been reported that naringin chalcone is rapidly converted to naringin (flavanones) by *CHI* and further synthesised into various flavonoids in most plants [51]. As mentioned above, no aurones or chalcones were detected in MLQS, perhaps due to the high expression of *CHI*.

During S1–S4, the expression levels of *NnFLS1* and *NnFLS2*, which catalyse flavonol synthesis from dihydroflavonols [52], were significantly higher in MLQS than in YGB, and there was no significant difference in the content of quercetin and its derivatives. However, the content of isorhamnetin and its derivatives was significantly higher in MLQS than in YGB. *AtOMT1* has been characterised as being involved in flavonol methylation to form isorhamnetin, supported by in vivo [53] and in vitro evidence in *Arabidopsis thaliana* [54]. In the present study, it is posible that quercetin in MLQS was rapidly converted to isorhamnetin by *NnOMT*. In addition, the expression of the three *NnOMTs* was higher in MLQS than in YGB, which confirmed our hypothesis to some extent. To date, a limited number of *OMT* genes have been functionally characterised in a particular organism due to the lack of sufficient substrate range and a high efficiency genetic transformation system [55]; these gaps have greatly limited the verification of *NnOMT*. The expression of the two putative *NnFLS* genes was significantly higher in MLQS than in YGB during S1–S4. Kaempferol(−D) content significantly increased from S2–S3 in the later flower colouration stages (Additional file 5: Figure S1), but was much lower in MLQS than in YGB during S3–S5. Previous studies have indicated that *FLSs* display variable substrate preferences and loose catalytic activities, which may contribute to their different isoforms [56–59]. *FtFLS1* in *Fagopyrum tataricum* was reported to be more active in converting dihydroquercetin to quercetin than in converting dihydrokaempferol to kaempferol [60]. *AtFLS1* was more effective in converting dihydrokaempferol to kaempferol than dihydroquercetin to quercetin in *A. thaliana* [61]. A study of *Citrus unshi* revealed that FLS had higher affinity to dihydrokaempferol than to dihydroquercetin. Therefore, we suggest that the *FLS* gene in the two lotus cultivars examined in this study may have different substrate preferences, with *NnFLS* genes more likely to catalyse the formation of kaempferol(−D) from dihydrokaempferol(−D) than the formation of quercetin(−D) from dihydroquercetin in the white cultivar YGB, whereas it has the opposite substrate preference in the yellow cultivar MLQS. This hypothesis is consistent with our metabolism data, which revealed significant differences between dihydroquercetin, isorhamnetin(−D), and kaempferol(−D) at S3–S5, when differences in flower colour between MLQS and YGB became obvious (Fig. 7). However, further experimental verification is needed. Pearson correlation analysis of flavonoid content and gene expression indicated that isorhamnetin(−D) was significantly correlated with *NnPAL1*, *NnF3’H1*, *NnF3’H2*, *NnFLS1*, and *NnOMT3* expression, whereas quercetin(−D) was significantly correlated with *NnPAL1* and *NnOMT3* expression. *NnPAL1* is an upstream gene that determines the activity of the entire pathway, and *NnF3’H1*, *NnF3’H2*, *NnFLS1*, and *NnOMT3* expression were consistent with our previous analysis to some extent. Flower colour formation is related to expression levels of key biosynthetic genes in the metabolic pathway. Nevertheless, upstream transcription factors of biosynthetic genes and post-transcriptional protein modification can also affect the expression and function of metabolic pathway genes [62, 63]. Thus, the expression of biosynthetic genes and metabolite levels are not necessarily linearly correlated.

## Conclusion

This study is the first analysis of the dynamics of primary and secondary metabolites during lotus flower colouration. We found that the shunting of isorhamnetin and kaempferol may result in different petal colours between the MLQS and YGB cultivars, because isorhamnetin(−D) content, which determines yellow colour, was significantly higher in MLQS than in YGB at nearly all flower colouration stages. Significant correlation was also detected between isorhamnetin(−D) content and the yellow flower colour parameter b*. Isorhamnetin(−D) may be a key pigment in the flavonoid pathway leading to differences between yellow and white flower colours in lotus. This result confirms the findings of a previous study of pigments in lotus cultivars with yellow and white flowers [12], but is inconsistent with those of a study that reported the contributions of aurone and chalcone flavonoids to bright yellow flower colour in other plants [21]. Therefore, we conclude that yellow flower colour in lotus may have a different mechanism compared with that in other plants. We produced a flowchart of the flavonoid pathway relevant to lotus flower colour, and further analysed the expression of key enzymes in this pathway in both lotus cultivars. Taken together, our results suggest that the substrate specificity of *NnFLS* genes and differential expression of *NnOMTs* are related to petal colour differences between MLQS and YGB. Future studies should further confirm the exact function of *NnFLSs* and *NnOMTs*, which may reveal the mechanism of yellow flower colour formation in lotus. This study will lay a foundation for further research on yellow petal colour breeding in lotus.

## Methods

### Plant materials

Two lotus cultivars with different flower colors, provided by China Lotus Research Center (Wuhan, China), were selected as the experimental materials. The detailed information of the two cultivars can be checked in the lotus flower cultivars in china [9]. Among external morphologies, petal color is the most obvious difference which distinguishes the two cultivars. MLQS is a cultivar owing luminous yellow petal color (YELLOW GROUP 10B of RHS 5th version, S5 in Fig. 1) when it is blossom, while the color of YGB is white (WHITE GROUP NN155D of RHS 5th version, S5 in Fig. 1). Each cultivar was planted in the water pool under the same cultivation conditions in the experimental base of Huazhong Agricultural University, Wuhan, China (30.51667°N, 114.31667°E). The samples were collected at five different coloration stages according to a continuous observation of flower bud coloration in whole growing season among 2015–2017. As for the yellow cultivar MLQS, In S1, the bud was light yellow green (WHITE GROUP 155C) with the longitudinal length of the bud reaching 1-2 cm; in S2, the bud turned light yellow (YELLOW-GREEN GROUP 150D) with 2–3 cm bud in the longitudinal length; in S3, the longitudinal length of the bud is 5–8 cm and the petals are pale yellow (YELLOW-GREEN group 150C); in S4, the longitudinal length of the bud is 8-10 cm with the yellow petals darken (YELLOW GROUP 2D) in color; S5 is the first day of blooming with a luminous yellow (YELLOW GROUP 10B) petal color (Fig. 1). The flower coloration process was divided into 5 stages for the white cultivar YGB correspondingly. In S1, the bud was white (YELLOW-GREEN group 154D) with the longitudinal length of the bud reaching 1-2 cm; in S2, the bud turned light yellow (YELLOW-GREEN GROUP 150D) with 2–3 cm bud in the longitudinal length; in S3, the longitudinal length of the bud is 5–8 cm and the petals are pale yellow (YELLOW GREEN GROUP 149B); in S4, the longitudinal length of the bud is 8-10 cm with the petals are white (WHITE GROUP NN155C) in color; S5 is the first day of blooming with a white (WHITE GROUP NN155D) petal color. The fresh petals were compared to Royal Horticultural Society Colour Chart (RHSCC) to describe the color at all the stages. Whole bud (S1-S2) and central part of middle-layer petals (S3-S4) were separated from the two cultivars at the five different developmental stages mentioned above (Fig. 1), and then sampled for primary and secondary metabolic analyses as well as RNA extraction. All samples were collected and immediately frozen in liquid nitrogen after measurement of flower color indices, and then stored at − 80 °C for further use.

Color indices of the central part of middle-layer petals were measured with a spectrophotometer CM-5 (Konica Minolta, Japan) and three parameters (CIE 1976) were applied to analyze the color. L* represents lightness, a* represents the red/green axis and b* suggests the yellow/blue axis [10, 64]. Chroma [C* = (a*2 + b*2) 1/2] and hue angle values (h*) [h* = Arctan (b*/a*)] are calculated by the value of a* and b*, representing the saturation and hue [64, 65]. The h* is a continuously fading hue circle to describe different colors with four special degree which described as red (0°/360°), green (180°), blue (270°), and yellow (90°) [65–67]. Color indices for each sample were measured with three biological repeats.

### Primary metabolic profiling by GC-MS

Non-targeted metabolite profiling was carried out by GC-MS using a modified method described by Yun et al. [29] and Tan et al. [68]. In summary, 200 mg flower samples were extracted in 2, 700 μl methanol and ribitol solution (300 μl, 0.2 mg ml^− 1^) was added as a quantification internal standard. The mixture was incubated firstly, then agitated, dried and derivatized. GC-MS analysis was performed by using a Thermo Trace GC Ultra, together with a Thermo Fisher TSQ 8000 Evo Triple Quadrupole mass spectrometer (Thermo Fisher Scientific, Waltham, MA, USA) as suggested by Yun et al. [29] and Tan et al. [68]. Metabolites were identified by searching in the NIST library. Quantification was based on the peak area ratios of the quantitation ions and the internal standard ribitol as described by Tan et al. [68].

### The secondary metabolic profiling by LC-MS

The secondary metabolic profiling was performed by LC-Q-TOF-MS using a modified method according to Yun et al. [29]. In detail, 100 mg freeze-dried powder was extracted with 80% methanol over night at 4 °C. Then the mixture was centrifuged and filtered. The metabolic profiling were performed using a Q-TOF 6520 mass spectrometer (Agilent Technologies, Palo Alto, CA, USA) coupled to a 1200 series Rapid Resolution HPLC system as described by Page et al. [69]. Raw data was processed by Agilent Mass Hunter Qualitative Analysis (version B. 04. 00, Aglient Technologies) and Mass Profiler Software (version B. 02.02, Aglient Technologies) based on Tan et al. [70]. Metabolites identification was performed by mass fragment analysis (MS/MS), comparing the accurate m/z values, the retention time, and the fragmentation patterns to available standards, or making comparisons to reported metabolites in literature and databases such as METLIN, MassBank, HMDB. Contents of metabolite identifications according to Sumner et al. [71]. Standards of Quercetin 3-O-hexoside and Quercetin were purchased from Sigma-Aldrich (USA). PCA analysis was performed by using the software Simca-P (Ver 11, Umetrics, Umea, Sweden).

### Expression profiling by qRT-PCR analysis

20 selected key homologs in flavonoid pathway were chosen based on the lotus annotation data [2] for validation using qRT-PCR on QuantStudio™ 7 Flex Real-Time PCR System (Applied Biosystems, Inc., Foster City, CA, United States) with gene-specific primers designed using Primer Premier software (version 5.0) (Additional file 1: Table S1). Total RNA of the 5 representative stages in flower coloration of MLQS and YGB were extracted using an EASYspin Plant RNA Kit (Aidlab, Beijing, China). The quality and quantity of the RNA was examined using 1% (w/v) agarose gel electrophoresis and a NanoDrop 2000 spectrophotometer (Thermo Fisher Scientific, Wilmington, DE, United States). Two microgram of total RNA was used for reverse transcription in a total volume of 20 μL using the 5X All-in-One Mastermix (AccuRT Genomic DNA removal Kit, Canada). The qRT-PCR reaction system was 10 μL, containing 5 μL SYBR Premix Ex Taq II (Tli RNaseH Plus) (2x), 0.2 μL ROX Reference Dye II (50x) (Takara, Dalian, China), 400 nM each primer and 1 μL 10-fold-diluted cDNA template. And the reactions were performed on the following program: 95 °C for 10 s; then 40 cycles of 95 °C for 10 s followed by annealing at 55 °C for 20 s and 72 °C for 20 s. Subsequently, the specificity of the individual PCR amplification was checked using a heat dissociation protocol from 55 °C to 95 °C following the final cycle of the PCR. Based on previous studies on lotus, actin was selected as an internal control [3, 10]. To ensure the reproducibility and reliability of the qRT-PCR results, three independent biological replicates and three technical replicates were arranged for each sample. Quantification of the relative expression of the genes was performed using the 2^-⊿⊿CT^ method, as described by Livak and Schmittgen [72].

### Statistical analysis

We performed ANOVA and Duncan’s multiple range test (*P* < 0.05 and *P* < 0.01) over the primary and secondary metabolic profiling of MLQS and YGB during flower coloration process. Significant levels were presented with lower case letters according to the results of Duncan’s multiple range test. The Student’s t-test was carried out for the differential metabolite analyses between MLQS and YGB with the *P*-value setting at 0.05 or 0.01. The results of qRT-PCR analyses were also determined by the Student’s t-test (P < 0.05) or (P < 0.01). All data analysis was performed using the SAS software (Version 8.0; SAS Institute, Cary, NC) with three biological repeats. Pearson correlation analysis was performed by the R software (Version × 64 3.5.0).

## Additional files

Additional file 1: **Table S1.** Primer sequences for qRT-PCR of flavonoid pathway structural genes. (DOC 14 kb)

Additional file 2: **Table S2.** Compounds identified in lotus during flower colouration stages measured by GC-MS. (XLS 11 kb)

Additional file 3: **Table S3.** GC-MS data of primary metabolites at five stages of lotus flower colouration. The data was normalized to ribitol and calculated as μg per g dry weight of petals. In the ANOVA and Duncan’s multiple range test results (Item A vs Item B), red and blue indicate comparing with Item A, Item B increased or decreased significantly (*p* < 0.05), and a, b, c, d indicated significant levels according to Duncan’s multiple range test (p < 0.05). (XLS 50 kb)

Additional file 4: **Table S4.** Secondary Metabolites detected at the five flower coloration stages of MLQS and YGB. Metabolites identification was performed by mass fragment analysis (MS/MS), matching their retention times and mass spectra to known standards, or making comparisons to reported metabolites in references and databases. Levels of metabolite identifications according to Sumner et al. Metabolomics (2007) 3:211–221. (XLS 43 kb)

Additional file 5: **Figure S1.** Heatmap showing secondary metabolites and their derivatives dynamics during flower coloration of **a** MLQS and **b** YGB. The proportion of each secondary metabolites and their derivatives in all periods from minimal to maximum are colored from blue to red. (PDF 1058 kb)

Additional file 6: **Table S5.** LC-MS data of secondary metabolites at five stages of lotus flower colouration. The data was normalized to lidocaine and calculated as μg per g dry weight of petals. In the ANOVA and Duncan’s test results (Item A vs Item B), red and blue indicate comparing with Item A, Item B increased or decreased significantly (p < 0.05), and a, b, c, d indicated significant levels according to Duncan’s multiple range test (p < 0.05). (XLS 83 kb)

Additional file 7: **Table S6.** Difference between MLQS and YGB on secondary metabolites during flower coloration. The data used for the Student’s t-test were the content of secondary metabolites (Table S5). In the Student’s t-test results (Item A vs Item B), red or blue indicate comparing with Item A, Item B increased or decreased significantly (p < 0.05) or highly significantly (*p* < 0.01). (XLS 24 kb)

Additional file 8: **Table S7.** Pearson correlation analyses between the Y/W primary metabolites and the secondary metabolites. * indicates significant correlation at *P* < 0.05 level, while ** indicates significant correlation at *P* < 0.01 level. Red and blue represent positive and negative correlations respectively. (XLS 13 kb)

## Data Availability

All data generated or analyzed during this study are included in this published article and its supplementary information files.

## Abbreviations

4’CGT: 4’-O-glucosyltransferase
4CL: 4-coumarate-CoA ligase
ANOVA: Analysis of variance
ANS: Anthocyanidin synthase
AS: Aureusidin synthase
C4H: Cinnamate 4-hydroxylase
cDNA: Complementary DNA
CHI: Chalcone isomerase
CHS: Chalcone synthase
DFR: Dihydroflavonol-4-reductase
EBGs: Early biosynthetic genes
F3’5’H: Flavonoid 3’: 5’-hydroxylase
F3’H: Flavonoid 3’-hydroxylase
F3H: Flavanone 3-hydroxylase
FLS: Flavonol synthase
GC-MS: Gas chromatography-mass spectrometry
HPLC: High performance liquid chromatography
LAR: Leucoanthocyanidin reductase
LC-MS: Liquid chromatography-mass spectrometry
LC-Q-TOF-MS: Liquid chromatography quadrupole time-of-flight mass spectrometry
metabolite(−D): Total content of metabolite and its derivatives
OMT: O-methyltransferase
PAL: Phenylalanine ammonia-lyase
PCA: Principal component analysis
qRT-PCR: Quantitative reverse-transcription polymerase chain reaction
TCA: Tricarboxylic acid
