# Supplementary material for: Differences in flavonoid pathway metabolites and transcripts affect yellow petal colouration in the aquatic plant Nelumbo nucifera
Source: BMC Plant Biol. 2019 Jun 24;19:277. doi: 10.1186/s12870-019-1886-8 (PMC6592004; doi:10.1186/s12870-019-1886-8)
Supplement: Supplementary file 1 — Table S1. Primer sequences for qRT-PCR of flavonoid pathway structural genes. (DOC 14 kb) [file 12870_2019_1886_MOESM1_ESM.doc]

**Table S1. Primer sequences for** **qRT-PCR of flavonoid pathway structural genes.**

| **Genes** | **Forward primer（****5'-3'）** | **Reverse primer (5'-3')** |
| --- | --- | --- |
| *Nn4CL* | ATAAAGTGTCGGTTGCATCGTT | TCAGTCATCCCATATCCCTGTC |
| *NnC4H* | AAGAGCTTGCCAGCGAAGTC | CGCCGTAGATTGTGAACACC |
| *NnCHS* | TAACAGCGAGCACAAAACCG | TCTTTTCCGAGCTTGGGCACTTCAA |
| *NnCHI* | GTTCTCGAATGGGAGCTTCATA | GCCGATTACGGATAACAGGTAG |
| *NnDFR* | TACAAGGGGAAACTCAAGCC | AGCAAAGAAACACCGAAAGG |
| *NnLAR* | TAGAGGGCAATCAGGGCATC | TGACGAGAGCGACTGTGGTA |
| *NnF3H1* | GCGATGGGGCTTGAAACA | CTGCGGGCATTTGGGATA |
| *NnF3H2* | ACCCTGGCACCATTACCCT | AACCTCCCGTTGCTCAGATAG |
| *NnF3'5'H* | GCCACAGCCATTCCGCA | CTTCTTCGTTACACGCCTTTTC |
| *NnF3'H1* | TTCCGCTCTTGAAGGTTTTG | TTCTCATTCGTCTTCACTCCCT |
| *NnF3'H2* | CTCAAGAAACAGGAAAGCCACA | GAGGGACGAGAAGGGGAACT |
| *NnF3'H3* | TGATGTCCGAGGTAACGATTTT | AGGGTGGCAGTCAAGAGGTG |
| *NnF3'H4* | ATGGCCCTTGATGGGGTAC | GATTGTGCGGCTGGAGAAC |
| *NnFLS1* | TGAAGGCTATGGCACGAAGA | TCCGTCACCTCCCTGTAAGAA |
| *NnFLS2* | CATACCCAGTGAGGTCATCCAG | TCTTGGGCCAGAACCTGTAGTTGAT |
| *NnPAL1* | TGACCAACCACGTCCAAAGT | CTCCAGGTGCCTCAAATCAA |
| *NnPAL2* | AGGGCACGGACAGTTATGGT | GGGCTCCTCCTTGTTTGGT |
| *NnOMT1* | TGGAGCATGTTGGAGGAGATAT | CGGTAACGCTTGGTAGCAGTT |
| *NnOMT2* | CGGTGGCGAATCAGTTGTT | CACTTAGCGATGGCTCTAGCAG |
| *NnOMT3* | CCAAGGGTAAACATCGGACTG | TGCTCGGAAATGGGGAAA |
| *ACTIN* | GCGTTCTGCCGTCTTCTAAA | CCCTCTTGGATTGTGCCTC |
