# Supplementary material for: Differences in flavonoid pathway metabolites and transcripts affect yellow petal colouration in the aquatic plant Nelumbo nucifera
Source: BMC Plant Biol. 2019 Jun 24;19:277. doi: 10.1186/s12870-019-1886-8 (PMC6592004; doi:10.1186/s12870-019-1886-8)
Supplement: Supplementary file 5 — Figure S1. Heatmap showing secondary metabolites and their derivatives dynamics during flower coloration of a MLQS and b YGB. The proportion of each secondary metabolites and their derivatives in all periods from minimal to maximum are colored from blue to red. (PDF 1058 kb) [file 12870_2019_1886_MOESM5_ESM.pdf]

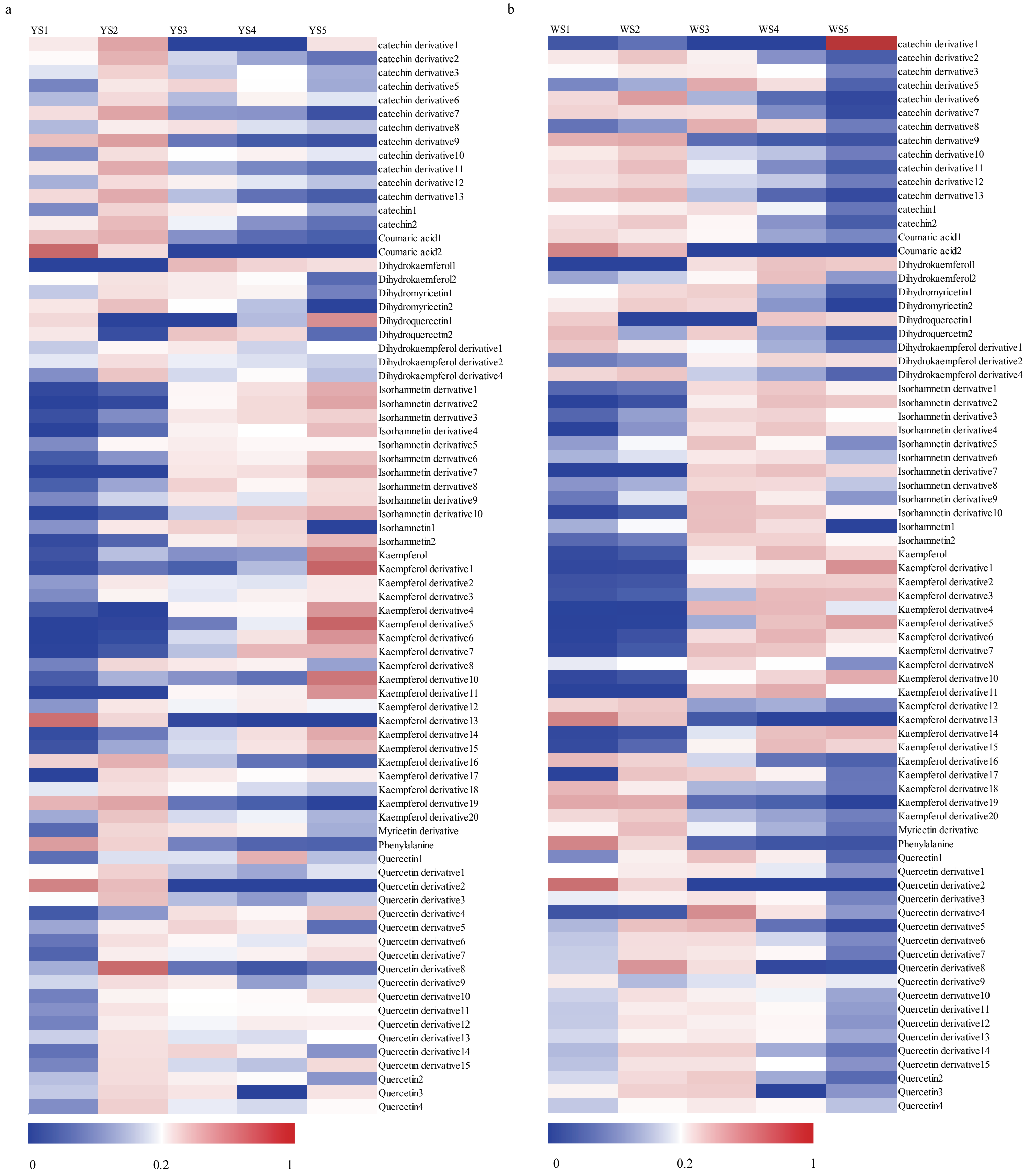
a
YS1
YS2
YS3
YS4
YS5
catechin derivative1
catechin derivative2
catechin derivative3
catechin derivative5
catechin derivative6
catechin derivative7
catechin derivative8
catechin derivative9
catechin derivative10
catechin derivative11
catechin derivative12
catechin derivative13
catechin1
catechin2
Coumaric acid1
Coumaric acid2
Dihydrokaemferol1
Dihydrokaemferol2
Dihydromyricetin1
Dihydromyricetin2
Dihydroquercetin1
Dihydroquercetin2
Dihydrokaempferol derivative1
Dihydrokaempferol derivative2
Dihydrokaempferol derivative4
Isorhamnetin derivative1
Isorhamnetin derivative2
Isorhamnetin derivative3
Isorhamnetin derivative4
Isorhamnetin derivative5
Isorhamnetin derivative6
Isorhamnetin derivative7
Isorhamnetin derivative8
Isorhamnetin derivative9
Isorhamnetin derivative10
Isorhamnetin1
Isorhamnetin2
Kaempferol
Kaempferol derivative1
Kaempferol derivative2
Kaempferol derivative3
Kaempferol derivative4
Kaempferol derivative5
Kaempferol derivative6
Kaempferol derivative7
Kaempferol derivative8
Kaempferol derivative10
Kaempferol derivative11
Kaempferol derivative12
Kaempferol derivative13
Kaempferol derivative14
Kaempferol derivative15
Kaempferol derivative16
Kaempferol derivative17
Kaempferol derivative18
Kaempferol derivative19
Kaempferol derivative20
Myricetin derivative
Phenylalanine
Quercetin1
Quercetin derivative1
Quercetin derivative2
Quercetin derivative3
Quercetin derivative4
Quercetin derivative5
Quercetin derivative6
Quercetin derivative7
Quercetin derivative8
Quercetin derivative9
Quercetin derivative10
Quercetin derivative11
Quercetin derivative12
Quercetin derivative13
Quercetin derivative14
Quercetin derivative15
Quercetin2
Quercetin3
Quercetin4
0
0.2
1
b
WS1
WS2
WS3
WS4
WS5
catechin derivative1
catechin derivative2
catechin derivative3
catechin derivative5
catechin derivative6
catechin derivative7
catechin derivative8
catechin derivative9
catechin derivative10
catechin derivative11
catechin derivative12
catechin derivative13
catechin1
catechin2
Coumaric acid1
Coumaric acid2
Dihydrokaemferol1
Dihydrokaemferol2
Dihydromyricetin1
Dihydromyricetin2
Dihydroquercetin1
Dihydroquercetin2
Dihydrokaempferol derivative1
Dihydrokaempferol derivative2
Dihydrokaempferol derivative4
Isorhamnetin derivative1
Isorhamnetin derivative2
Isorhamnetin derivative3
Isorhamnetin derivative4
Isorhamnetin derivative5
Isorhamnetin derivative6
Isorhamnetin derivative7
Isorhamnetin derivative8
Isorhamnetin derivative9
Isorhamnetin derivative10
Isorhamnetin1
Isorhamnetin2
Kaempferol
Kaempferol derivative1
Kaempferol derivative2
Kaempferol derivative3
Kaempferol derivative4
Kaempferol derivative5
Kaempferol derivative6
Kaempferol derivative7
Kaempferol derivative8
Kaempferol derivative10
Kaempferol derivative11
Kaempferol derivative12
Kaempferol derivative13
Kaempferol derivative14
Kaempferol derivative15
Kaempferol derivative16
Kaempferol derivative17
Kaempferol derivative18
Kaempferol derivative19
Kaempferol derivative20
Myricetin derivative
Phenylalanine
Quercetin1
Quercetin derivative1
Quercetin derivative2
Quercetin derivative3
Quercetin derivative4
Quercetin derivative5
Quercetin derivative6
Quercetin derivative7
Quercetin derivative8
Quercetin derivative9
Quercetin derivative10
Quercetin derivative11
Quercetin derivative12
Quercetin derivative13
Quercetin derivative14
Quercetin derivative15
Quercetin2
Quercetin3
Quercetin4
0
0.2
1
